# Current News

**Published:** 1892-12

**Authors:** 


### Current News.

### THE WORLD'S COLUMBIAN DENTAL CONGRESS, TO BE HELD IN CHICAGO, AUGUST 17 TO 27, INCLUSIVE, 1893.

[THE following abstract is taken from the general circular of the Finance Committee of the World's Dental Congress.—ED.]

"It has been voted that the membership-fee shall be ten dollars. To be collected only from residents of the United States.

"It has been voted 'that the membership shall consist of legally qualified and reputable dentists (as defined in the Code of Ethics of the American and Southern Dental Associations) residing in the United States, and all foreign dentists regularly qualified by the laws of the countries from which they come, and such other scientific persons as may be invited by the Committee on Invitations' (Committee No. 11).

"It will be seen that the doors are open to every reputable dentist in the United States. All will be welcome except those who are illegal practitioners in States having laws, or who are violators of the Code of Ethics. Should any such join, or attempt to join, and it is known, the name of such applicant will be referred to the Membership Committee. It is advised that no subscription involving any right of membership be received by the State Finance Committees from such as are known to be ineligible to membership.

"The General Finance Committee submitted to the Executive Committee the following plan, and it was unanimously voted that such plan should constitute the 'rule of action':

"*Resolved,* That the General Finance Committee be authorized to guarantee to contributors who would be eligible to membership as follows:

"*First.* That every contributor of ten (10) dollars shall receive the Transactions if he does not attend, but will be expected to pay the membership-fee of ten (10) dollars in addition, should he attend the Congress.

"*Second.* That every contributor of twenty (20) dollars shall receive the Transactions of the Congress if he does not attend, and if he attends and joins the Congress, his receipt for contribution shall be accepted by the Treasurer as full payment of the membership-fee of ten (10) dollars.

"*Third.* That every contributor of thirty (30) dollars or upward shall have all the advantages of the contributor of twenty (20) dollars, and in addition shall receive free the Commemorative Medal which will be struck.

"From the foregoing it will be seen that the solicitor for contributions can assure each contributor of ten dollars or upward that he will receive a substantial return, whether he attends or not.

"To accomplish all this work will demand a large amount of money. The items of stationery, printing, and postage will call for considerable ready cash, which must be advanced by those doing the work, in addition to their generous and gratuitous labor, or be furnished by the prompt, liberal, and patriotic contributions of the dentists of the whole country. Careful estimates place the amount to be contributed in advance of the Congress at thirty thousand dollars.

"It is judged by all who are conversant with the work done up to the present time that this Congress will mark an epoch in dental progress, and be the largest in numbers and most valuable in results of any dental meeting ever held.

"The General Executive Committee feels that a great trust has been committed to it, which it will endeavor to manage in a broad and liberal spirit.

"The General Finance Committee has perfect confidence that the profession will respond promptly and generously to its call for contributions to insure the success of the Congress and the honor of American dentistry.

"The receipt which the Treasurer, Dr. J. S. Marshall, will mail to each contributor will have printed on it the conditions before mentioned as part of the contract between the Congress and each contributor."

### RECENT PATENTS.

A LIST of recent patents, reported specially for the INTERNATIONAL DENTAL JOURNAL:

481,740.—Dental Burring Tool. R. G. Stambrough, New York, N. Y. Filed January 20, 1892.

482,520.—Portable Dental Chair. F. H. Field, New Orleans, La. Filed June 20, 1891.

482,558.—Dental Drill. Louis Maillard, Galt, Canada. Filed March 31, 1892.

482,552.—Dental-Tool Sterilizer. W. G. Flanders, New York, N. Y. Filed February 9, 1892.

483,807.—Dentist's Chair. William A. Johnston and Arthur W. Browne, Prince's Bay, N. Y., assignors to the S. S. White Dental Manufacturing Company, Philadelphia, Pa. Filed October 31, 1889.

484,287.—Dental Instrument. John C. Blair, Louisville, Ky. Filed March 2, 1892.

484,747.—Dental Chair. Levi Stuck, Bad Axe, Mich., assignor to Aaron P. Gould, John C. Skelton, and Albert Hœffer, Canton, Ohio. Filed September 4, 1890.

485,010.—Dental Instrument. Charles W. Jones, St. Paul, Minn. Filed February 4, 1892.

Trade-Marks. 21,882.—Dental Rubber. Excelsior Rubber Works, New York, N. Y. Filed September 13, 1892. Essential feature, the word "Ideal."

---

### AMERICAN DENTAL SOCIETY OF EUROPE.

THE Eighteenth Annual Meeting of the American Dental Society of Europe was held at Basel, August 1, 2, and 3. The following officers were elected:

President, Dr. L. C. Bryan, of Basel; Vice-President, Dr. J. H. Spaulding, of Paris; Treasurer, Dr. Charles J. Monk, of Wiesbaden; Secretary, Dr. Chas. W. Jenkins, of Zürich.

On account of the large number of members who intend to visit Chicago in 1892, the next meeting was appointed for August, 1894, at Geneva.

CHAS. W. JENKINS,
*Secretary.*

### CALIFORNIA STATE BOARD OF DENTAL EXAMINERS.

At the last annual meeting, August 4, of the California State Board of Dental Examiners, twelve applicants were examined, of which two were successful, and the following officers elected: President, J. L. Asay, M.D., San José; Secretary, J. D. Hodgen, D.D.S., San Francisco.

J. D. Hodgen,
*Secretary.*

---

### CALIFORNIA STATE DENTAL ASSOCIATION.

At the Twenty-third Annual Meeting of the California State Dental Association, held at San Francisco, July 19 to 22, the following officers were elected for the ensuing year: President, W. J. King, San Francisco; First Vice-President, L. A. Teague, San Francisco; Second Vice-President, I. W. Hays, Grass Valley; Third Vice-President, J. P. Parker, Santa Cruz; Recording Secretary, L. Van Arden, San Francisco; Corresponding Secretary, Chas. E. Post, San Francisco; Treasurer, T. N. Iglehart, San Francisco.

Chas. E. Post,
*Corresponding Secretary.*

